# The effect of age on CD4+ T-cell recovery in HIV-suppressed adult participants: a sub-study from AIDS Clinical Trial Group (ACTG) A5321 and the Bone Loss and Immune Reconstitution (BLIR) study

**DOI:** 10.1186/s12979-021-00260-x

**Published:** 2022-01-03

**Authors:** Jingxian Chen, Kehmia Titanji, Anandi N. Sheth, Rajesh Gandhi, Deborah McMahon, Ighovwerha Ofotokun, M. Neale Weitzmann, Kristina De Paris, Julie B. Dumond

**Affiliations:** 1 Eshelman School of Pharmacy, University of North Carolina at Chapel Hill, Chapel Hill, NC USA; 2 Present Address: Merck Co. & Inc., Rahway, NJ USA; 3 Division of Endocrinology & Metabolism & Lipids, Department of Medicine, Emory University School of Medicine, Atlanta, GA USA; 4 Division of Infectious Diseases, Department of Medicine, Emory University School of Medicine, Atlanta, GA USA; 5 Department of Infectious Disease, Massachusetts General Hospital and Ragon Institute, Boston, MA USA; 6 Department of Medicine, University of Pittsburgh, Pittsburgh, PA USA; 7 Atlanta VA Medical Center, Decatur, GA USA; 8 Department of Microbiology & Immunology, School of Medicine, University of North Carolina at Chapel Hill, Chapel Hill, NC USA; 9 Present Address: UNC at Chapel Hill, Chapel Hill, NC USA

**Keywords:** HIV, Aging, Immune recovery, Pharmacodynamic modeling, Nonlinear mixed effects modeling

## Abstract

Older age could be a risk factor for suboptimal CD4+ T-cell recovery in HIV-infected patients despite successful viral suppression. However, evaluation of this effect could be confounded by age-related immune processes such as decreased thymus output, increased immune activation and exhaustion. Here, we established a semi-mechanistic population model simultaneously describing naïve and memory CD4+ T-cell trajectories in 122 participants. Covariate analysis accounting for immune activation showed that older age was significantly associated with faster apparent elimination rate of the naïve T-cells. In addition, female sex predicted slower apparent elimination rate of memory T-cells. Simulations showed that the median maximal CD4+ T-cell count on ART treatment was 593 cells/μL (IQR 442-794) in patients aged 50 years or above and 738 cells/μL (IQR 548-1002) in patients aged 18-35 years. The differences in the percentage of subjects achieving sufficient immune reconstitution (CD4+ T-cell count> 500 cells/μL) between the two age groups were 15, 21 and 26% at year 1, 4 years and steady state, respectively, suggesting that advanced age may have a greater impact on long-term CD4+ T-cell recovery.

**Supplementary Information:**

The online version contains supplementary material available at 10.1186/s12979-021-00260-x.

## Introduction

Older HIV-infected patients achieve viral suppression similar to younger ones under ART [1]. In terms of immunological response, a negative effect on CD4+ T-cell recovery in older patients was reported, although a few studies found similar immunological outcomes in older and younger HIV-infected patients. Table 1 summarizes five studies with inconsistent findings regarding the influence of age on CD4+ T-cell recovery. These studies were selected because of their relatively large sample sizes of studied patients and long follow-up period [2–6].

**Table 1:** Controversial findings on aging influence on CD4+ T-cell recovery

Citation	Sample size	Study length	The effect of age
Kaufmann et al., JAMA, 2003 [3]	2235	4 years	Older age (> 40 years) ↓ CD4+ T-cell change by 39 cells/μl over 48 months
Moore et al., CID, 2007 [5]	655	6 years	Older age (> 45 years) ↓ CD4+ cell change by 60 cells/μl over 6 years
Li et al., JAIDS, 2011 [4]	614 men	5 – 12 years	Older age (≥50 years) ↓ CD4+ T-cell counts by 59 cells/μl over 5-12 years
Greenbaum, AIDS, 2008 [2]	906	4 years	Nonsignificant difference in time to increase by 50 cells/μL or mean increase in CD4+ T-cells from baseline between > 50 years vs. < 40 years
Wright et al., HIV medicine, 2013 [6]	3378	≥ 5 years	Nonsignificant difference in CD4+ T-cell increase rate in > 50 years vs. others

Inconsistency in study design and data analyses, such as the definitions of CD4+ T-cell recovery and different age stratifications, may explain the discrepant findings. In addition, aging may influence CD4+ T-cell recovery through different mechanisms and sometimes in opposing directions, and the overall effect of aging may be masked. Aging is known to decrease thymic output which leads to the decreased de novo synthesis of naïve CD4+ T-cells [7, 8]. As a potential off-setting effect, aging could extend the half-life of naïve T-cells [9]. Additionally, age-related factors present in chronic HIV infection, such as immune activation and immune exhaustion, could be potential confounders in these analyses, as they were also reported to be influential factors of impaired CD4+ T-cell recovery [10–12]. However, in most past studies, these factors were separately studied, and therefore the interactions among these factors were not evaluated.

**Fig. 1:**
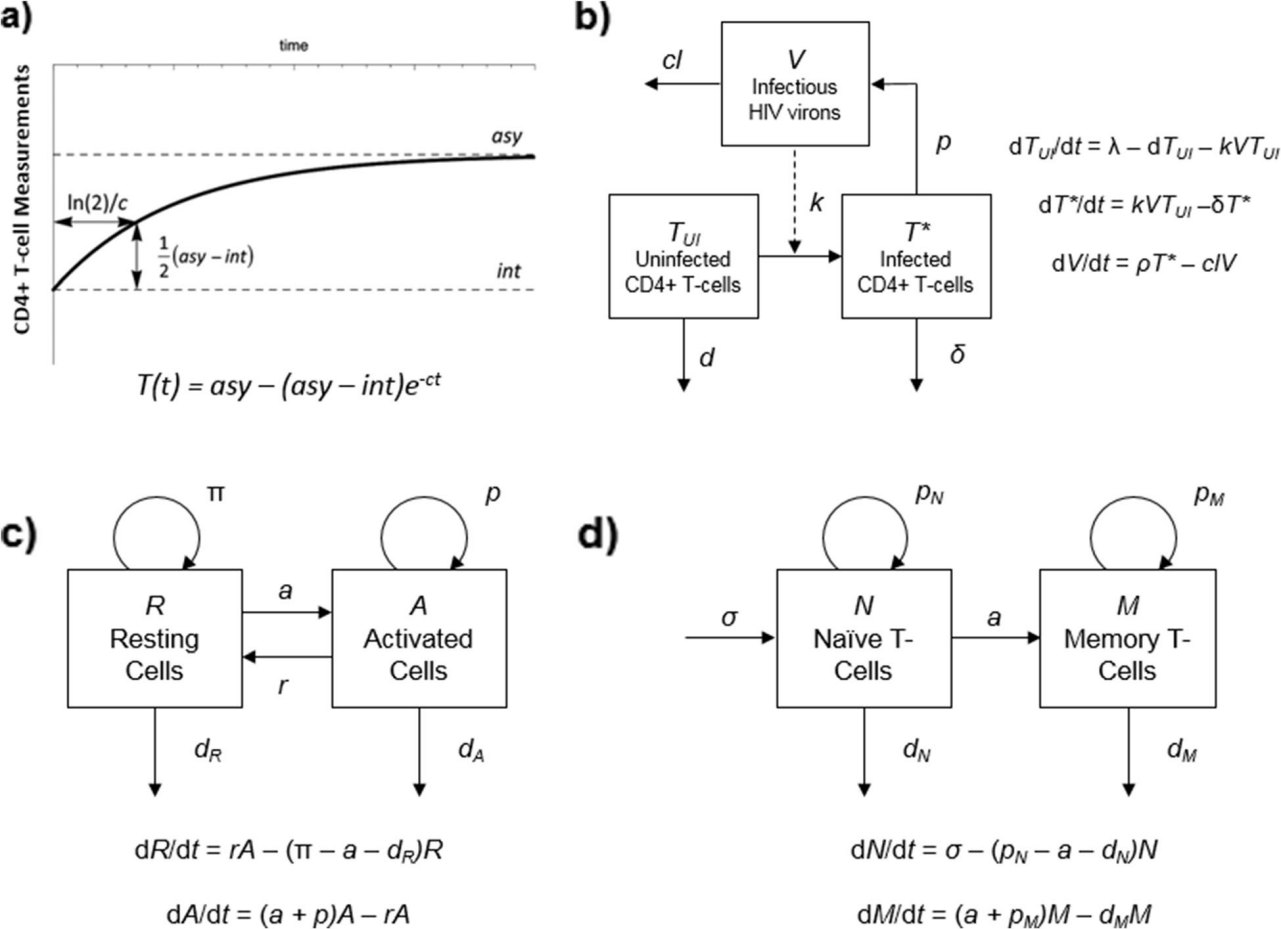
Different Modeling of T-cell Dynamics. **a** Simple exponential model of total CD4+ T-cells. ***T***: total T-cell measurement, ***int***: initial CD4 + T-cell measurement, ***asy***: asymptotic or long-term CD4+ T-cell measurement on treatment, ***c***: cell elimination rate constant, ***ln(2)/c***: time to achieve half asymptotic CD4+ T-cell measurement. **b** Joint viral and T-cell dynamic model in HIV infection. ***TUI***: uninfected T-cells, T*: infected Tcells, ***V***: infectious HIV virons, ***λ***: production rate of uninfected T-cells, ***d***: death rate constant of uninfected T-cells, ***k***: viral infection rate constant, ***δ***: death rate constant of infected T-cells, ***ρ***: new viron production rate constant, ***cl***: viron clearance rate constant. **c** and **d** Mechanistic models of T-cell homeostasis. ***R***: resting T-cells, ***A***: activated T-cells, ***a***: activation rate constant, ***r***: rate constant of reversion to resting state, ***π***: resting cells proliferation rate constant, d: death rate constant, ***p***: proliferation rate constant, ***N***: naïve T-cells, ***M***: memory T-cells, ***σ***: naïve T-cell production rate

Semi-mechanistic mathematical modeling approaches are particularly useful in describing longitudinal data, by relating the dynamics of CD4+ T-cells to the rates of physiological processes. Therefore, the evaluation of the relevant factors on the process of interest is possible. Several models have been proposed to describe CD4+ T-cell recovery in HIV-infected patients on ART (summarized in Fig. 1). A simple exponential function is the most basic CD4+ T-cell recovery model, but lacks insight into the various CD4+ T-cell subtypes [7, 13] (Fig. 1a). CD4+ T-cell dynamics in a joint viral and T-cell dynamic model in HIV infection (Fig. 1b) describes non-infected and infected T-cells [14], and can also include compartments for latently, persistently and defectively infected T-cells [15]. These models provide a mechanistic framework for investigations of pharmacokinetic-pharmacodynamic analysis of antiretrovirals (ARVs), however, they do not address long-term T-cell homeostasis. Also, not all the parameters of these models are able to be estimated precisely when fitted to the data, and they often rely on values from the literature derived under different conditions or different patient populations. Mechanistic models describe the system in a more physiologically relevant way and, therefore, may be easier to extrapolate to long-term outcomes.

Two types of theoretical models have been proposed to study the mechanisms of T-cell homeostasis during HIV infection and after ART. One type of model consists of resting and activated cells populations [16, 17] (Fig. 1c), and cell labeling methods were used to estimate dynamic parameters which usually only reflect the status of the time of experiment. The other type of model includes naïve and memory T-cells populations [18] (Fig. 1d), and is generally used to investigate the dynamics of thymic output and proliferation of naïve T-cells using biomarker data in short-term studies [8, 19, 20]. Fitting regular CD4+ T-cell clinical data using these two types of models are often difficult due to complexity in model structure. Without describing variability, analysis of physiological/biological meaningful factors in these theoretical models is challenging; measurements of CD4+ T-cell counts have high inter-subject variability. In many retrospective cohort studies, the follow-up period also widely varies for each patient. When immune biomarkers are included, the sparsity in the measurement of these markers relative to the intensive total CD4+ T-cell data further increases complexity. Given these potential pitfalls, mixed results among previous analyses are not surprising.

Population modeling provides a solution for these challenges by accommodating variety in data collection. Furthermore, it evaluates difference sources of variability efficiently in one analysis. In this work, we aimed to use semi-mechanistic population modeling approaches to evaluate the role of aging on CD4+ T-cell recovery in HIV-infected patients on effective ART with adjustment of multiple immunological factors to overcome the inherent challenges and limitations of the existing models.

## Results

### Study participant characteristics and available data

In total, the current analysis included data from 102 HIV participants from ACTG A5321, and 20 participants with measurements at all longitudinal time points from the control arm of the Bone Loss and Immune Reconstitution (BLIR) study [21]. The studied subjects had a median age of 39 years at treatment initiation, with a range of 18-64 years. 80% of the subjects were male. The median CD4+ T-cell baseline was 256 cells/μL. Subjects from the BLIR study had significantly lower baseline CD4+ T-cell counts compared to those from ACTG (*P* < 0.0001), implying that these subjects were more immunocompromised compared to the subjects enrolled in ACTG A5321. The BLIR study also had more past/current smokers than ACTG (*p* = 0.014). Patient characteristics are summarized in Table 2. Additionally, in terms of ART in the ACTG participants, the median/interquartile range (IQR) age was similar across regimen type. For those receiving an NNRTI + NRTI, the median/IQR age was 38 (31, 44); for PI + NRTI, 39 (30, 48); and 42 (28-48) years for those receiving RAL + NRTI.

**Table 2:** Demographics of study populations

Baseline characteristics		ACTG cohort (*n* = 102)	BLIR study cohort (*n* = 20)	Total (*n* = 122)
**Age (years)**		39 (18 – 64)	37 (30 – 51)	39 (18 – 64)
**Sex**	Female	21 (21%)	3 (15%)	24 (20%)
	Male	81 (79%)	17 (85%)	98 (80%)
**Race**	Caucasian Non-Hispanic	51 (50%)	5 (25%)	56 (46%)
	African American Non-Hispanic	19 (19%)	14 (70%)	33 (27%)
	Hispanic	30 (29%)	1 (5%)	31 (25%)
	Other	2 (2%)	0	2 (2%)
**BMI**		25.7 (18.6 – 47.2)	23.3 (18.9 – 35.5)	25.4 (18.6 – 47.2)
**Prior Smoking History**	Yes	52 (51%)	16 (80%)	68 (56%)
	No	50 (49%)	4 (20%)	54 (44%)
**CD4+ T-cells (cells/mm**^**3**^**)**		288 (8 – 980)	72.5 (3 – 268)	256 (3 – 980)
**Viral load (log**_**10**_**RNA copies/mL)**		4.61 (2.28 – 5.99)	5.3 (2.15 – 6.14)	4.67 (2.15 – 6.14)
**ART Regimen**	NNRTI + NRTI	42 (41%)	0	42 (34)
	PI + NRTI	39 (38%)	20 (100%)	59 (48%)
	RAL + NRTI	19 (19%)	0	19 (16%)
	Other	2 (2%)	0	2 (2)
**HBV Infection**	Positive	1 (1%)	No data	No data
	Negative	101 (99%)	No data	No data
**HCV Infection**	Positive	3 (3%)	No data	No data
	Negative	99 (97%)	No data	No data

Continuous variables are presented as median (range). Categorical variables are presented as count (percentage)

*ACTG* AIDS Clinical Trials Group, *BLIR* Bone Loss and Immune Reconstitution, *BMI* body mass index, *HBV* hepatitis B virus, *HCV* hepatitis C virus

The data available for the current analysis contained 2581 and 175 total CD4+ T-cell counts from the ACTG cohort (over up to 15 years) and from the BLIR study (over 144 weeks), respectively. The T-cell subtype biomarker, CD45RA, was available in 340 and 80 samples for the computation of naïve T-cell counts in the ACTG and BLIR study, respectively. In addition, 364 CD38/HLA-DR measurements of activated CD4+ and CD8+ T-cells were available in the ACTG study. The BLIR study provided for the following biomarkers: 81 CD31 (a marker of thymic output), 77 CD38/HLA-DR and 81 PD-1/TIM-3 (markers of T cell exhaustion) measurements on CD4+ T-cells, 77 CD38/HLA-DR and 79 PD-1/TIM-3 measurements on CD8+ T-cells. CD4+ T-cell counts and biomarker versus time profiles are presented in Supplementary Figs. 1 and 2, respectively.

### Structural CD4+ T-cell recovery model

**Fig. 2:**
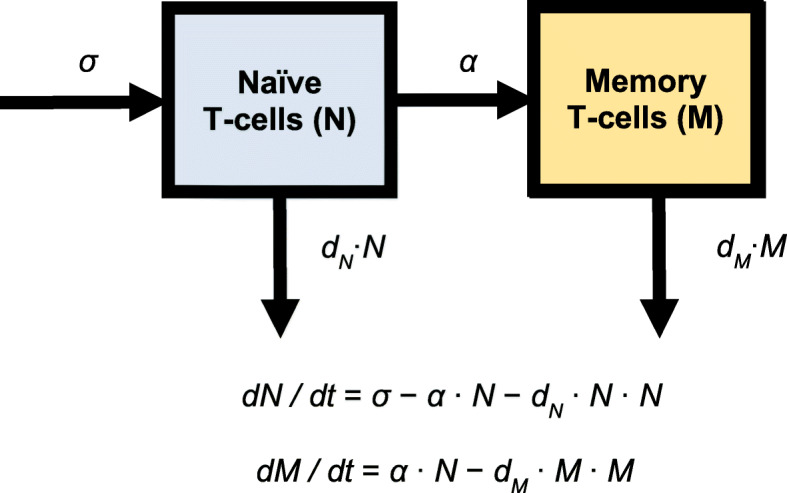
Structural Model Schematic. **σ**: production rate of naïve T-cells, ***α***: activation rate constant of naïve T-cells that acquire a memory phenotype, ***dN***: naïve T-cell death rate constant, ***dM***: memory T-cell death rate constant, **N**: naïve T-cell number, **M**: memory T-cell number

The schematic of the structural model and the differential equations are shown in Fig. 2. Naïve (defined as CD45RA) and total T-cell counts were simultaneously analyzed in the structural model, which consisted of the naïve and memory T-cell compartments. The total CD4+ T-cell count was the sum of the naïve and memory CD4+ T-cell count. The production of naïve T-cells was described using a zero-order process (σ). The conversion of naïve to memory T-cells was described using a first-order process (α). The proliferation process was not able to be separated from the elimination process, and therefore combined into the elimination term. The cell density dependent process best described cell elimination (d_N_ • N for naïve T-cell and d_M_ • M for memory T-cell). The baselines of naïve (baseline_N_) and memory (baseline_M_) T-cells were estimated. Inter-individual variabilities (IIVs) were added on all parameters, with correlations between the IIVs of σ and α. Residual errors were described using a mixed proportional and additive model.

The structural model described both the ACTG and BLIR data reasonably well, as evaluated by the diagnostic plots and individual model fit (Supplementary Fig. 3). All the parameters were precisely estimated with percent relative standard error (RSE%) < 30%, with reasonably low shrinkage of IIVs, indicating the use of this structural model for the further covariate analysis was appropriate. The parameter estimates of the structural model established from only the ACTG data and the combined data, are summarized in Supplementary Table 1. According to the diagnostic plots of stratified by age (Supplementary Fig. 3), there was no obvious bias in the model across different age groups.

### Covariate analysis in the population model

The pre-selected parameter-covariate relationships for each parameter based on general additive modeling (GAM) analyses and investigator interest are summarized in Table 3. Hepatitis B (HBV) and Hepatitis C (HCV) infection were removed from the candidate list due to the low frequency of co-infected subjects (< 3%). The stepwise covariate modeling (SCM) analysis found advanced age at ART initiation was a significant covariate for larger d_N_, suggesting that aging was a risk factor for poor CD4+ T-cell recovery. Since d_N_ was a combination of cell proliferation and death (d_N_ = rate constant of cell death – rate constant of cell proliferation), this relationship might indicate that aging was associated with slower cell proliferation or faster cell death or a combination of both. Female sex was associated with slower d_M_, and similar to the above case, suggests that females had faster cell proliferation or slower cell death or both, as this is also a combined parameter. In addition, higher viral load at ART initiation predicted lower naïve and memory T-cell baseline. Higher immune CD4+ T-cell activation was associated lower memory T-cell baseline. Table 4 summarizes the SCM analysis in the model.

**Table 3:** Pre-selected parameter-covariate relationships

Parameters	Covariates to be included in SCM
σ [(cell/μL)*year^−1^]	Age, ART class, viral load, sex, BMI, race
d_N_ (year*cell/μL)^−1^	Age, ART class, viral load, sex, smoking status
α (year^−1^)	Age, CD4+ T-cell immune activation, CD8+ T-cell immune activation, smoking status, viral load
d_M_ (year*cell/μL)^−1^	Age, ART class, CD4+ T-cell immune activation, CD8+ T-cell immune activation, sex, smoking status
Baseline_N_ (cell/μL)	Age, CD4+ T-cell immune activation, CD8+ T-cell immune activation, viral load, sex
Baseline_M_ (cell/μL)	Age, CD4+ T-cell immune activation, CD8+ T-cell immune activation, race, sex

**σ**: production rate of naïve T-cells, ***α***: activation rate constant, ***d***_***N***_: naïve T-cell death rate constant of naïve T-cells that acquire a memory phenotype, ***d***_***M***_: memory T-cell death rate constant, **baseline**_**N,**_**baseline**_**M**_: naïve or memory T-cell count at ART initiation

*ART* antiretroviral therapy, *BMI* body mass index, *SCM* stepwise covariate modeling

**Table 4:** Stepwise covariate analysis

Parameter	Covariate	*P* value		Coefficient
**Forward Addition (α = 0.05)**				
σ [(cell/μL) • year^− 1^]	No significant covariates	NA		NA
d_N_ (year • cell/μL)^− 1^	Age	< 0.001		0.0467
d_M_ (year • cell/μL)^− 1^	Sex (Female)	0.0039		−0.510
	Smoking Status (No prior smoking history)	0.019		0.573
α (year^− 1^)	Age	0.046		0.0203
	%Activated CD4+ T-cells	0.042		0.228
baseline_N_ (cell/μL)	Viral load (log_10_RNA/mL)	< 0.001		−0.636
	%Activated CD4+ T-cells	0.015		−0.446
baseline_M_ (cell/μL)	Viral load (log_10_RNA/mL)	< 0.001		−0.388
	%Activated CD4+ T-cells	< 0.001		−0.396
	%Activated CD8+ T-cells	0.0091		0.249
**Backward Elimination (α = 0.01)**		*P* value	△IIV (%)	
σ [(cell/μL) • year^−1^]	No significant covariates	< 0.01	NA	NA
d_N_ (year • cell/μL)^−1^	Age		−9.6	0.0389
d_M_ (year • cell/μL)^−1^	Sex (Female)		−5.0	−0.597
α (year^−1^)	No significant covariates		NA	NA
baseline_N_ (cell/μL)	Viral load (log_10_RNA/mL)		−20	−0.648
baseline_M_ (cell/μL)	Viral load (log_10_RNA/mL)		−17.3	−0.422
	%Activated CD4+ T-cells		−8.3	−0.378

**σ**: production rate of naïve T-cells, ***α***: activation rate constant of naïve T-cells that acquire a memory phenotype, ***d***_***N***_: naïve T-cell death rate constant, ***d***_***M***_: memory T-cell death rate constant, **baseline**_**N**_: naïve T-cell count at ART initial, **baseline**_**M**_: memory T-cell count at ART initial, IIV: inter-individual variability, NA: not applicable. Parameter-covariate relations are described using a linear function, e.g. *P*_*i*_ = *P* • (1+ *b* * age), with *P*_*i*_ as the individual value, *P* as the population value and *b* as the covariate coefficient. Immune activation, presented as %CD38+/HLA-DR+ on the cells, are log-transformed

### Final model evaluation

Parameter estimates of the final model are presented in Table 5. The model point estimates agreed with the bootstrap median estimates and were precisely estimated with reasonably low %RSEs and narrow bootstrap 95% confidence intervals. The diagnostic plots for total and naïve CD4+ T-cells (Supplementary Fig. 4) showed that stratified by age (< 35 years, 35- < 50 years, > 50 years), the observed data were well predicted, with no obvious bias in the model. The prediction-corrected visual predictive checks demonstrated a good performance of the model in predicting the overall data and also stratified by age, with the observed percentiles well captured by the predicted percentiles and corresponding 95% confidence intervals (Supplementary Fig. 5 for VPCs of all data and Supplementary Fig. 6 for VPCs stratified by age).

**Table 5:** Final parameter estimates

Parameter (units)	Estimates (RSE%)			Bootstrap estimates	
	Point estimates	RSE (%)		Median	95% CI
σ [(cell/μL) • year^− 1^]	703	19		700	(484, 1006)
d_N_ (year • cell/μL)^− 1^	0.0410	22		0.0410	(0.0255, 0.0661)
d_M_ (year • cell/μL)^−1^	0.000432	13		0.000435	(0.000314, 0.000559)
α (year^−1^)	1.10	13		1.11	(0.85, 1.45)
baseline_N_ (cell/μL)	30.5	13		29.8	(20.3, 38.9)
baseline_M_ (cell/μL)	211	10		210	(173, 250)
**Covariate Effects**					
VL-Baseline_M_	−0.422	20		−0.424	(−0.552, − 0.174)
IACD4-Baseline_M_	− 0.378	18		−0.375	(− 0.435, − 0.138)
VL-Baseline_N_	− 0.648	3.8		− 0.645	(− 0.733, − 0.477)
Age-d_N_	0.0389	37		0.0394	(0.0124, 0.0514)
Sex-d_M_ (Female)	−0.48	25		−0.485	(− 0.664, − 0.212)
**Inter-individual variability (CV%)**			**Shrinkage (%)**		
σ	116	7.3	7.5	115	(99.7, 136)
d_N_	63.7	20	41	63.4	(28.9, 82.7)
d_M_	59.9	14	38	57.7	(37.3, 78.2)
α	125	9.8	5.8	123	(97.3, 148)
baseline_N_	135	8.9	9.3	137	(105, 165)
baseline_M_	85.8	9.5	32	83.6	(68.5, 101)
cor(ω^2^σ ~ ω^2^α)	−0.802	7.2	–	−0.805	(−0.913, −0.679)
**Residual variability (CV%/SD)**					
Total, proportional	16.3	3.3	6.9	16.3	(15.2, 17.3)
Total, additive	31.2	22	18	31.7	(15.4, 44.9)
Naive, proportional	42.1	6.6	15	41.5	(35.7, 46.7)
Naive, additive	2.03	14	23	2.01	(1.19, 2.68)

**σ**: production rate of naïve T-cells, ***α***: activation rate constant of naïve T-cells into memory phenotype, ***d***_***N***_: apparent naïve T-cell elimination rate constant, ***d***_***M***_: apparent memory T-cell elimination rate constant, **baseline**_**N**_: naïve T-cell count at ART initial, **baseline**_**M**_: memory T-cell count at ART initial, **VL**: HIV viral load (log_10_RNA/mL), **IACD4**: CD4+ T-cell activation

T-cell activation is defined by %CD38/HLA-DR, and is analyzed as log-transformed values

*CV* coefficient of variance, *CI* confidence interval, *RSE* relative standard error

### Simulations of the effect of age on total CD4+ T-cell counts

**Fig. 3:**
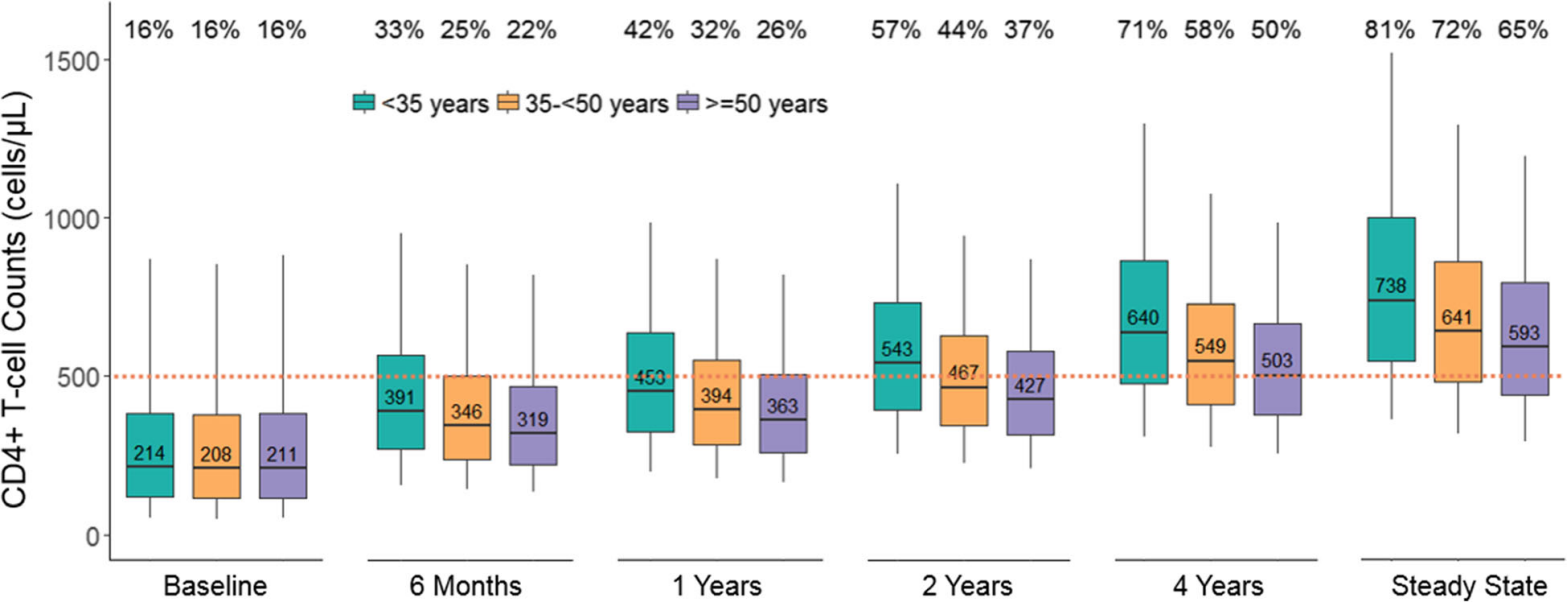
Simulated CD4+ T-Cell Recovery after Antiretroviral Therapy by Age Group. CD4+ T-cell count at 5th, 25th, 50th, 75th and 95th of the simulations (represented by the whiskers and horizontal lines of the boxplots) of each age group at time after treatment are presented. The median CD4+ T-cell counts are indicated for each group. Dotted line at 500 cells/μL is chosen as the index for CD4+ T-cell count recovery. The percentages of the simulated subjects achieving immune recovery are indicated for each group on the top of the figure

The simulated total CD4+ T-cell counts and the percentages of simulated subjects demonstrating immune reconstitution (IR), defined as a total cell count of ≥500 cells/μL, at different times after ART treatment are summarized in Fig. 3. Twenty years after treatment initiation was considered steady state as it was sufficiently long for the CD4+ T-cell recovery of all the studied patients to reach the criteria for steady state given the predicted individual parameters (data not shown). At the time of ART initiation, the simulated CD4+ T-cell counts from the established model were similar across age groups (around 210 cells/μL in median). At year 4 after ART treatment, median CD4+ T-cell counts of all age group reached 500 cells/μL. At steady state, median CD4+ T-cell counts were 739 (IQR 548-1002), 641 (IQR 481-860) and 593 (IQR 441-794) cells/μL for simulated patients aged < 35 years, 35- < 50 years and ≥ 50 years, and the percentage of subjects showing sufficient IR were 81, 72 and 65%, respectively. Comparing the younger (< 35 years) and older (≥50 years) age groups, the differences of median CD4+ T-cell counts between the two groups were 93, 137 and 145 cells/μL at year 1, 4 years and steady state, respectively, suggesting that advanced age had a greater impact on impaired CD4+ T-cell recovery over extended periods of time.

### Preliminary analysis of the effects of thymus output and immune exhaustion

**Fig. 4:**
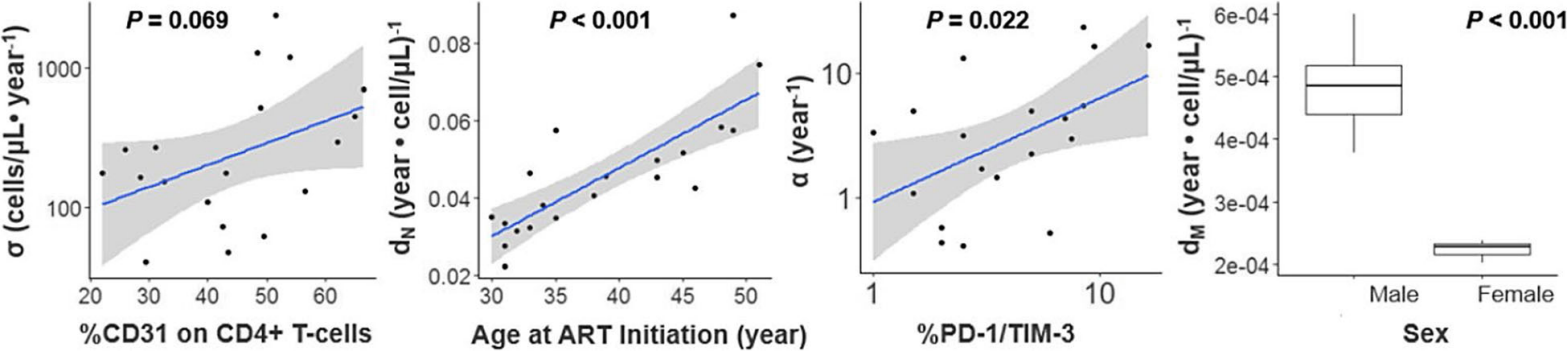
Post Hoc Analysis of CD4+ T-Cell Dynamic Parameters. σ: production rate of naïve T-cells, α: activation rate constant of naïve T-cells that acquire a memory phenotype, dN: naïve T-cell death rate constant, dM: memory T-cell death rate constant. %CD31 on naïve CD4+ T-cells defines thymus output of naïve CD4+ T-cells, %PD-1/TIM-3 coexpression defines immune exhaustion. P values from the linear regression for each relation are indicated

Thymic output and immune exhaustion were explored using the available data in BLIR study (relevant data were not available from the ACTG cohort). As expected, the production rate of naïve T-cells (*σ*) was significantly associated with %CD31 expressed on CD4+ naive T-cells, a biomarker of thymus output (*P* < 0.001). For the apparent elimination rate constant of naïve T-cells (*d*_*N*_), age and activation level on CD8+ T-cells were statistically significant covariates when tested separately, while age was the only significant predictor in the multivariate regression model (*p* < 0.001). Higher levels of immune exhaustion were significantly associated with a faster activation rate constant of naïve T-cells (*α*) (*P* = 0.022). Sex was a significant covariate for the apparent elimination rate constant of memory T-cells (*d*_*M*_) (*p* < 0.001). When adjusted for these effects, age was not a significant predictor of either *σ, α* or *d*_*M*_. The covariate effect of age in this analysis was consistent with the findings in the model. Visual relationships discussed above are shown in Fig. 4. Linear regression analyses are summarized in Supplementary Table 2.

## Discussion

In this study, we evaluated the impact of aging, in terms of chronological age, on the CD4+ T-cell recovery in HIV-infected patients with successful viral suppression. To our knowledge, this is the first semi-mechanistic population model describing longitudinal CD4+ T-cell dynamics under ART treatment, incorporating the evaluation of important immune biomarkers. Using T-cell subtype biomarker data, we divided the total T-cell kinetics into naïve and memory T-cell dynamics, enabling a more mechanistic evaluation of aging effects on CD4+ T-cell recovery. Our analysis highlights that older age may be a predictor of suboptimal immune recovery by influencing CD4+ naïve T-cell homeostasis. Among the three age groups (18- < 35, 35- < 50 and ≥ 50), the percentages of simulated participants achieving sufficient immune reconstitution were 42, 32 and 26% at the first year after ART treatment, and 81, 72 and 65% at steady state, respectively. The differences of CD4+ T-cell recovery between age groups increased over time, indicating that the negative impact of older age on CD4+ T-cell recovery becomes more profound with longer follow up. We also observed that thymus output, immune activation and immune exhaustion affected immune reconstitution.

The model describes naïve and total CD4+ T-cell simultaneously. According to the current data, the naïve T-cell dynamics are described using a zero-order input and a cell density-dependent elimination process. The memory T-cells are converted from the naïve T-cells with a first-order process and are eliminated following a cell density-dependent elimination process. Our data did not support the estimation of the proliferation process, even with different variations in parameterization. We chose not to fix the proliferation parameter to the literature values due to the large variation and uncertainty in this estimate; previous studies using isotope labeling methods measured the proliferation rate for total T-cells without differentiating naïve and memory T-cells as we did [16, 20]. In studies quantifying thymus output and proliferation, instead of fitting the data, the proliferation rate was studied in an exploratory manner by simulating different CD4+ T-cell dynamic scenarios, therefore those values were theoretical and no validation was performed [8, 20]. Therefore, we chose to define the apparent elimination in our model as a combination of the cell proliferation and of cell death, with the rate proportional to the number of cells in the compartment.

The model was developed using data from two cohorts, which represented long-term (ACTG) and short term (BLIR) CD4+ T-cell recovery profiles, respectively. The model was primarily developed from the ACTG model with a larger sample size (*n* = 102), and the model was able to well-describe the data from the BLIR study (*n* = 20). To minimize the variability in measuring the biomarkers, the gating strategies from the ACTG study were applied to the BLIR study data as much as possible. In the final model, different residual error models were used for ACTG data (proportional) and BLIR data (proportional + additive), however, the proportional error residuals were similar between the two studies and therefore combined in one term for both total and naïve T-cell observations.

The stepwise covariate analysis in the model suggested that age at ART initiation was positively associated with the apparent naïve T-cell elimination rate constant, the parameter describing both naïve cell proliferation and death. The literature findings support the negative association between age and cell proliferation. It is possible that aging is associated with cell replicative senescence as a result of age-dependent DNA demethylation [22]. A study that directly measured cell proliferation rate of CD4+ T-cells in mice using a DNA labeling method showed that there was a two-fold drop of proliferation rates of T-cells in thymus with aging in two different mice strains [23]. On the other hand, a previous study showed a 3-fold increase in the half-life of naïve T-cells in aged mice compared to younger ones, suggesting an extended cell life span, or slower cell death, as protective mechanism for naïve T-cell pool in elderly, which is contrary to the positive relationship between age and cell death. Therefore, we propose that aging impairs CD4+ T-cell recovery by decelerating naïve T-cell proliferation. Our analysis showed that a 10-year increase in age is associated with a 4% increase in the apparent naïve T-cell elimination rate constant. In terms of CD4+ total T-cell counts, simulation showed that steady state median CD4+ T-cell counts decrease by 13 and 20% in a patient aged 40 years and 50 years, respectively, compared to a patient aged 30 years. However, these changes are not profound compared to the large variability in CD4+ T-cell counts (~ 30% coefficient of variance) [24], and their clinical significance is likely minimal. The current simulation does not account for the reduced thymus function and impaired immune response in older individuals (e.g. reduced responses to vaccination). The T-cell receptor (TCR) repertoire undergoes contraction with age, and the reduced TCR repertoire, combined with the inadequate T-cell reconstitution after ART initiation, may amplify the defects observed in immune recovery in older HIV-infected subjects. Further investigation on CD4+ T-cell functions is warranted to better evaluate the clinical importance of these findings.

In our analysis, female sex was favorably associated with CD4+ T-cell recovery as it reduced the apparent memory T-cell elimination rate by 48%. As discussed above, the interpretation of this relationship could be multidirectional. Though the mechanism has not been clearly elucidated, it has been hypothesized that female hormones provide a beneficial environment for CD4+ T-cell homeostasis, both in the general [25] and the HIV-infected population [26]. Our simulation showed that at steady state, the median change in CD4+ T-cell counts from baseline was 592 cells/μL (IQR 474-704) in female and 416 cells/μL (IQR 348-463) in male, with a ~ 42% increase in females compared to males. Our analysis associated higher HIV viral load at ART initiation with lower naïve and memory T-cells. Moreover, a one-log increase in viral load had a greater impact on naïve T-cell baseline compared to that of memory T-cells (decrease by 65% versus 42%). In addition, higher level of CD4+ T-cell activation was associated with lower memory T-cell baseline, which might be a result of increased activation-induced T-cell apoptosis [27].

Thymic output is the mechanism for naïve T-cell generation. The current post hoc analysis with the 20 subjects in the BLIR study suggested a strong association between CD31 expression on naïve T-cells and naïve T-cell production rate (*P* < 0.001). This finding is consistent with the well-delineated relationship between thymus involution and aging in the literature [7, 8]. The activation level on CD4+ T-cell was nominally associated with the activation rate constant of the conversion from naïve to memory T-cells, however, this effect was not detected in the final model, probably due to the adjustment for immune activation on the baseline memory T-cells. However, we found that a higher activation rate was significantly associated with higher immune exhaustion (Fig. 4). Immune exhaustion is characterized by a progressive loss of T-cell functions, and commonly develops during viral-persistence in HIV infection as a result of chronic immune activation and persists even in individuals fully suppressed on ART [28]. Our analysis suggested that the higher level of immune exhaustion was unfavorable for naïve T-cell restoration as a result of increased activation rate.

Our study has several strengths and provides a new way to approach longitudinal CD4+ recovery data. Using a semi-mechanistic population model, we were able to determine the effect of aging on naïve T-cell dynamics, and to our knowledge, this is the first time this relationship was observed in HIV-infected patients. Though the exact mechanism is not known, our study may stimulate future investigations into the impact of aging on T-cell dynamics in HIV-infected patients. We included multiple immune parameters and their changes in relation to age, data that informed the potential roles of these parameters in CD4+ T-cell dynamics, and allowed us to correct for potential confounding effects. We performed the analysis using data from two studies with long-term and short-term follow-up, which effectively informed different phases of CD4+ T-cell recovery that do not exist in a single study. The data from the ACTG study cover the CD4+ T-cell trajectory with the longest study period reported in the literature thus far (up to 15 years), which provided a robust perspective of the steady-state CD4+ T-cell counts in the HIV-infected population on ART and supported predictions of the long-term trajectory with more confidence. The intensive biomarker data available in the BLIR study added to the granularity of early stage of immune recovery when CD4+ T-cell reconstitution undergoes the greatest changes.

On the other hand, our analysis also has some limitations. Our analysis is retrospective, and the missing information in some patients, such as thymus output and immune exhaustion biomarkers, prevented the evaluation of these factors in the model. Also, a large portion of our studied population was under 50 years old, and we did not have many patients with more advanced age (above 65 years) at ART initiation. The pre-specified biomarkers for division of naïve and memory T-cell were CD45RA+/CCR7+, however, due to assay issues, the CCR7 data were not available. We did not include a common marker of immune aging, the CD4:CD8 ratio, due to potential issues with collinearity with the model structure. Our study sample size was relatively small, which limited the generalizability of our conclusions; and these data largely came from patients initiating treatment at lower CD4 counts with older ART regimens. Current HIV treatment guidelines now recommend treatment initiation immediately upon diagnosis regardless of CD4+ T-cell count, with more potent, better tolerated regimens; therefore, these finding may not be generalizable to aging patients who have initiated treatment in the last 5 years. As long-term immune recovery data from people living with HIV treated earlier with newer regimens become available, this model can be further refined and adapted.

In summary, we developed a population model describing longitudinal CD4+ T-cell recovery in HIV-infected patient with successful viral suppression under ART. Our analysis revealed the association between older age and impaired naïve T-cell recovery and the negative effect of aging on long-term total CD4+ T-cell reconstitution. Our preliminary analysis shed lights on the role of immune exhaustion on CD4+ T-cell dynamics as well as the association between higher thymus output and naïve T-cell generation. Further analysis will require more subjects in which these biomarkers were measured to validate our findings.

## Methods

### Studied population and data

The data for this analysis were obtained from two sources, the AIDS Clinical Trials Group Protocol 5321 (ACTG P5321) and the Bone Loss and Immune Reconstitution (BLIR) study conducted at Emory University.

The ACTG P5321 is a longitudinal cohort study which investigates the differences and changes in HIV reservoirs over time. Participant enrollment and data collections were described in the original publication [29]. Briefly, participants in this cohort were treatment-naïve and were on continuous ART during follow-up. All the participants had undetectable plasma HIV-1 RNA levels (< 50 copies/mL) at 48 weeks of ART and at all subsequent time points. T-cell HIV-1 levels, T-cell activation, T-cell subtype and inflammatory biomarkers were available in the paired plasma or peripheral blood mononuclear cell (PBMC) samples obtained before ART and at year 1, 4 and 6-15 on treatment. Total CD4+ T-cell counts were collected every 8-16 weeks during the follow-up. The data usage for the current analysis was approved by both ACTG and the UNC Biomedical Institutional Review Board after an exempt review. Data retrieved included the following:

1. Longitudinal total CD4+ and CD8+ T-cell counts,
2. Immune biomarkers: CD4+ T-cell subtype (%CD45RA), CD4+ and CD8+ T-cell immune activation (%CD38+/HLA-DR+) before ART and at year 1, 4 and 6-15 on treatment (depending on the availability),
3. Initial ART regimens: NNRTI-based (FTC/TDF/EFV, 3TC/ZDV/EFV, ABC/3TC/EFV, ABC/3TC/ZDV/EFV), PI-based (ABC/3TC/RTV/ATV, FTC/TDF/RTV/ATV, FTC/TDF/RTV/DRV), INSTI-based (FTC/TDF/RAL) or others (3TC/ZDV/EFV/NFV, EFV/RTV/LPV)
4. Pre-treatment characteristics: age, BMI, race/ethnicity, sex, plasma HIV-1 RNA level, prior smoking history (yes/no), co-infection with HBV or HCV (positive/negative).

The BLIR study is a prospective, randomized trial aiming to investigate the association between ART-associated bone loss and immune reconstitution. All participants received ARV/RTV 300/100 mg + TDF/TFC 300/200 mg once daily for HIV treatment. Paired plasma and PBMC samples were collected for skeletal and immune profiling at pre-defined time points from week 0 to 144 on treatment. Biomarkers defining T-cell subtype, thymus output, immune activation and immune exhaustion were measured at week 0, 12, 24 and 48 on treatment. Total CD4+ T-cell counts were measured every 4-24 weeks. The original study was approved by the Emory University Institutional Review Board (NCT01228318). The data usage for the current analysis was approved by both the collaborators and the UNC Biomedical Institutional Review Board after an exempt review. Data retrieved included the following:

1. Longitudinal total CD4+ T-cell counts and HIV viral load at week 0,4, 16, 24, 36, 48, 72, 96, 120 and 144,
2. Immune biomarkers: thymus output (%CD31+), CD4+ T-cell subtype (%CD45RA), CD4+ and CD8+ T-cell immune activation (%CD38+/HLA-DR+), CD4+ and CD8+ T-cell immune exhaustion (%PD-1+/TM-3+),
3. Demographics: age, gender ethnicity, race, smoking status (ever smoked, prior or current, cigarettes per day, years smoked), weight, height, BMI

### Population modeling

Modeling of the CD4+ T-cell count data was performed using NONMEM 7.4 (ICON Development Solutions, Hanover, MD). Data management, graphical analysis, post-modeling analysis and standard statistical analysis were conducted in R (version 3.4.3, r-project.org). Pirana (version 2.9.2) was used for model management and NONMEM output visualization. The ADVAN6 subroutine and first-order conditional estimation with interaction (FOCE-I) method were used for model development.

### Immune biomarker handling

Naïve CD4+ T-cell count was calculated by multiplying %CD45RA+ of CD4+ T-cells and total CD4+ T-cell count at the corresponding time point. Memory CD4+ T-cell count was described as the difference between total and naïve CD4+ T-cell count. The levels of thymus output, immune activation and immune exhaustion were evaluated as %CD31+, %CD38+/HLA-DR+ and %PD-1+/TIM-3+, respectively. Thymus output was measured for CD4+/CD45RA T-cells. Immune activation and immune exhaustion were measured for CD4+ and CD8+ T-cells.

### Structural model establishment

**Fig. 5:**
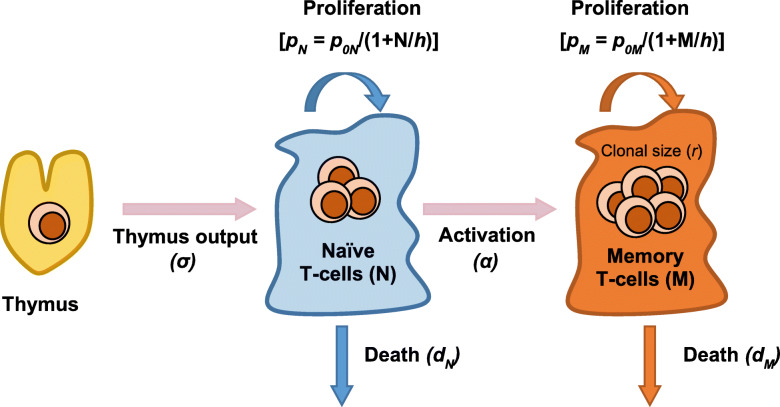
Cartoon Illustration of the Establishment of the CD4+ T-Cell Pool. **N**: naïve T-cell pool size, **M**: memory T-cell pool size, **σ**: Thymus production of naïve T-cells, ***α***: activation of naïve T-cells that acquire a memory phenotype, ***dN***: naïve T-cell death, ***dM***: memory T-cell death, ***pN***: naïve T-cell proliferation, ***p0N***: maximum proliferation of naïve T-cells, ***p0M***: maximum proliferation of memory T-cells, ***h***: pool size at half maximum proliferation; ***r***: clonal size resulting from activation of a single naive T cell

The model framework was based on the model proposed by Hanzenberg et al. [18] to describe the establishment of the CD4+ T-cell pool in children, both healthy and with HIV infection. The model consists of two cell populations, naïve and memory CD4+ T-cells. The thymus output contributes to the naïve T-cell production. The increase of naïve T-cell pool also depends on naïve T-cell proliferation. The expansion of memory T-cell pool is a result of the activation of naïve T-cells and the proliferation of memory T-cells. Both naïve and memory T-cells undergo cell death which decreases the T-cell pool size. The model illustration is shown in Fig. 5.

The structural model was initially developed using the ACTG data due to its relatively larger sample size and longer study period. A zero-order process was used to describe the naïve T-cell production from thymus output. The proliferation process was tested with a first-order and a capability-limited process. The capability-limited rate constant was parametrized as below:

1 $$ \boldsymbol{p}=\boldsymbol{p}\mathbf{0}/\left(\mathbf{1}+\raisebox{1ex}{$\boldsymbol{n}$}\!\left/ \!\raisebox{-1ex}{$\boldsymbol{h}$}\right.\right) $$

where *p* denotes the proliferation rate constant, *p*_*0*_ denotes the maximum proliferation rate constant, n denotes the cell number and *h* denotes the cell number at half maximum proliferation rate constant.

The cell activation and elimination was tested with a first-order and a cell density-dependent process. The cell density-dependent elimination rate was linear associated with the cell number, denoted as d*n, where d denotes elimination rate constant and n denotes cell number.

Inter-individual variability (IIV) was assumed to be normally distributed and exponentially related to the population parameters. Proportional, additive and combined proportional-additive error models were tested for residual variability. The correlation between the residual error of total and naïve T-cells was explored. Model discrimination was determined by change in OFV, using the likelihood ratio test (α = 0.05), diagnostic plots and precision of parameter estimates.

The structural model was initially developed from ACTG data [30], and was then used to fit the combined dataset from the two studies. The data fitting was examined by regular diagnostic plots.

### Covariate analysis

Covariate candidates included chronological age, gender, race, BMI, smoking status, pre-ART viral load, initial ART class, initial ART regimes, co-infection of HBV or HCV and levels of immune activation. Immune activation data, presented as percent CD38+/HLA-DR+ on T-cells, were log-transformed to achieve normality and stabilize the covariate model. Pre-selection of parameter-covariate relations were based on biologic plausibility, previous reports and the general additive modeling (GAM) analysis performed on the model-predicted parameters. GAM was performed using R package Xpose4 (version 4.5.3, Sourceforge). Model discrimination of GAM was based on Akaike Information Criteria (AIC). Covariate analyses in the non-linear mixed-effects model were conducted using the SCM procedure in PsN (version 4.6.0). The significant levels for forward addition and backward elimination were 0.05 and 0.01, respectively.

For BLIR data, candidate covariates also included levels of thymus output and immune exhaustion. Due to the small portion of subjects from BLIR study in the combined data (16%), the covariate analysis of these two factors was performed on the post hoc estimates derived from the final model from the SCM analysis, using linear regression methods. The effect of age was re-evaluated. A *P* < 0.05 was defined as statistically significant.

### Simulations of the effect of age on immune recovery

Simulations of total T-cell counts in three age groups, < 35 years, 35- < 50 years and ≥ 50 years, were performed at year 0, 0.5, 1, 2, 4 and at steady state after ART. Reaching the CD4+ T-cell recovery steady state was determined as the difference between the CD4+ T-cell counts at two consecutive years less than 1% of the former simulated cell count. The age of 50 years was the documented definition of “older” patients in the field of HIV treatment, and the cut-off of 35 years was chosen based on the equality of subjects in both groups in the studied population. For each age group, 100 simulated subjects aged within the corresponding intervals were created. All the characteristics of the simulated subjects were randomly sampled from the distribution of those of the studied population. Simulations were conducted 200 times. The percentages of simulated CD4+ T-cell counts reaching 500 cells/μL were calculated. Total CD4+ T-cell counts greater than 500 cells/μL was chosen as an indication for sufficient immune reconstitution in HIV-infected patient on treatment [31].

### Standard statistical analysis

Comparisons of continuous and categorical demographic variables were performed using Wilcoxon-Mann-Whitney test and Fisher’s exact test, respectively in R (version 3.4.3, r-project.org).

## Supplementary Information

**Additional file 1.**
