# Supplementary material for: The effect of age on CD4+ T-cell recovery in HIV-suppressed adult participants: a sub-study from AIDS Clinical Trial Group (ACTG) A5321 and the Bone Loss and Immune Reconstitution (BLIR) study
Source: Immun Ageing. 2022 Jan 3;19:4. doi: 10.1186/s12979-021-00260-x (PMC8722153; doi:10.1186/s12979-021-00260-x)
Supplement: Supplementary file 1 — Additional file 1. [file 12979_2021_260_MOESM1_ESM.docx]

**Supplemental Material**


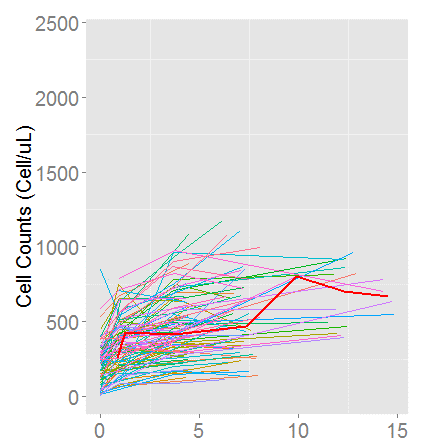

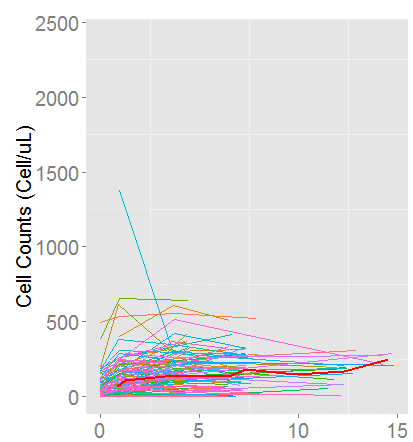

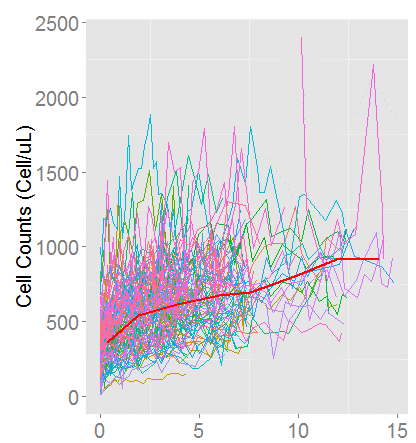


**CD4 T-Cell Counts (Cell/µ+L)**

**Memory CD4+ T-cells**

**Naive CD4+ T-cells**

**Total CD4+ T-cells**

**ACTG 5321 Study**


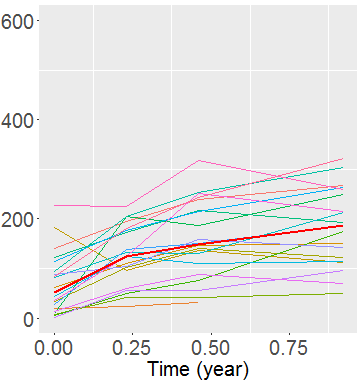

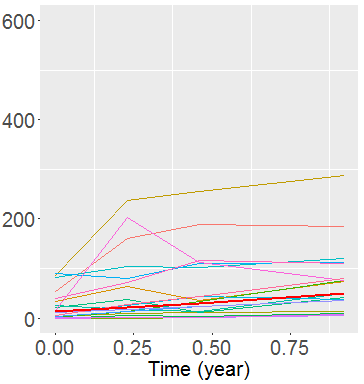

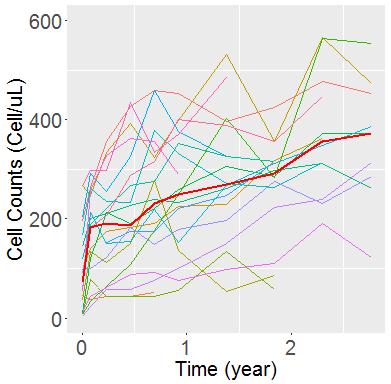


**Memory CD4+ T-cells**

**Naive CD4+ T-cells**

**Total CD4+ T-cells**

**CD4+ T-cell Counts (Cell/µL)**

**Time (year)**

**BLIR Study**

CD4+ T-cell counts versus time profiles from different participants are presented in different colors. Red lines represent the median CD4+ T-cell counts profiles.

Supplemental Figure 1. CD4+ T-Cell Trajectory in Participants from ACTG and BLIR Study.


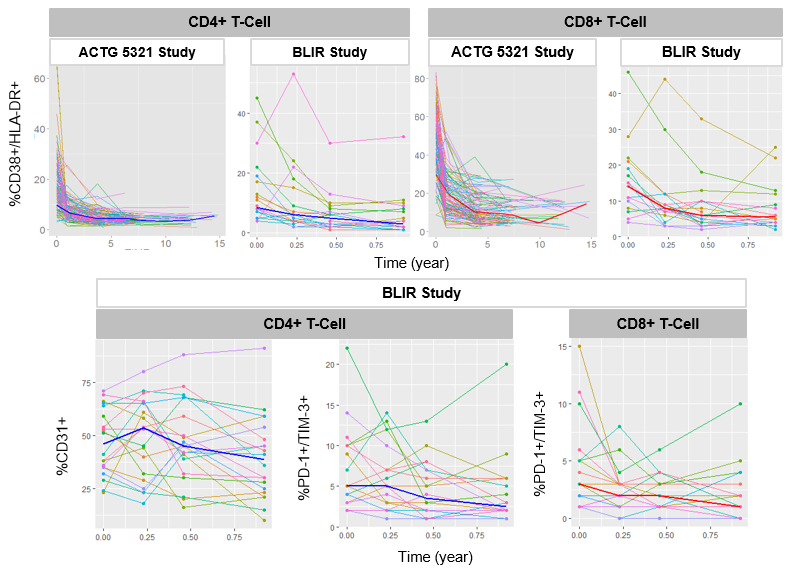


Different participants are presented in different colors. Immune biomarkers are presented as expression percentage on immune cells. Blue and red lines represent the median percentage of immune biomarker expression on CD4+ and CD8+ T-cells, respectively.

Supplemental Figure 2. Immune Biomarker Levels versus Time Profiles in Participants from ACTG and BLIR Study.

**Supplemental Table 1. Parameter Estimates of the Structural Model**

| **Parameter (Units)** | **Estimates (RSE%)** |
| --- | --- |
| σ [(cell/µL)*year^-1^] | 720 (18) |
| d_N_ (year*cell/µL)^-1^ | 0.0403 (25) |
| d_M_ (year*cell/µL)^-1^ | 0.000365 (14) |
| α (year^-1^) | 1.08 (14) |
| baseline_N_ (cell/µL) | 25.7 (21) |
| baseline_M_ (cell/µL) | 150 (10) |
| **Inter-individual variability (CV%) [Shrinkage%]** | |
| σ | 117 (7) [7.9] |
| d_N_ | 70.9 (14) [37] |
| d_M_ | 64.0 (13) [37] |
| α | 127 (10) [5.8] |
| baseline_N_ | 150 (9) [8.9] |
| baseline_M_ | 98.9 (8) [3.1] |
| **Residual variability (CV%)** |  |
| Total, proportional | 16.3 (3) [6.9] |
| Total, additive | 31.5 (24) [18] |
| Naive, proportional | 42.1 (7) [15] |
| Naive, additive | 2.05 (14) [22] |

**σ**: production rate of naïve T-cells, ***α***: activation rate constant of naïve T-cells that acquire a memory phenotype, ***d_N_***: naïve T-cell death rate constant, ***d_M_***: memory T-cell death rate constant, **baseline_N_**: naïve T-cell count at ART initial, **baseline_M_**: memory T-cell count at ART initial, **CV**: coefficient of variance, **RSE**: relative standard error.

Supplemental Figure 3. Diagnostics of Structural Model.

a)-d) and e)-h) are observation versus individual prediction, observation versus population prediction, observation (DV) vs. individual prediction (IPRED), with both an identity line (solid) and a Loess line (dashed); observation (DV) vs. population prediction (PRED), with an identity line (solid) and a Loess line (dashed); conditional weighted residual (CWRES) vs. time; conditional weighted residual (CWRES) vs. population prediction plots for total CD4+ T-cells and naïve T-cells, respectively. i)-l) are histograms of the inter-individual variability of σ, α, d_N_ and d_M_. σ: production rate of naïve T-cells, α: activation rate constant of naïve T-cells that acquire a memory phenotype, d_N_: naïve T-cell death rate constant, d_M_: memory T-cell death rate constant. The age groups are presented as different colors.


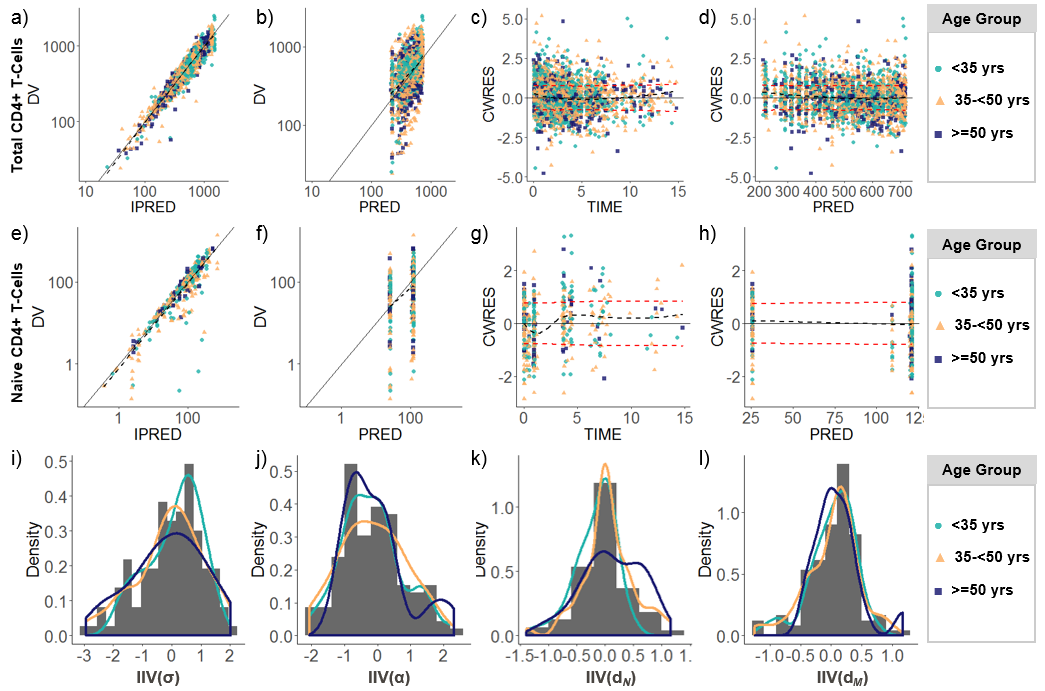


a)-d) and e)-h) are observation versus individual prediction, observation versus population prediction, observation (DV) vs. individual prediction (IPRED), with both an identity line (solid) and a Loess line (dashed); observation (DV) vs. population prediction (PRED), with an identity line (solid) and a Loess line (dashed); conditional weighted residual (CWRES) vs. time; conditional weighted residual (CWRES) vs. populations prediction plots for total CD4+ T-cells and naïve T-cells, respectively.


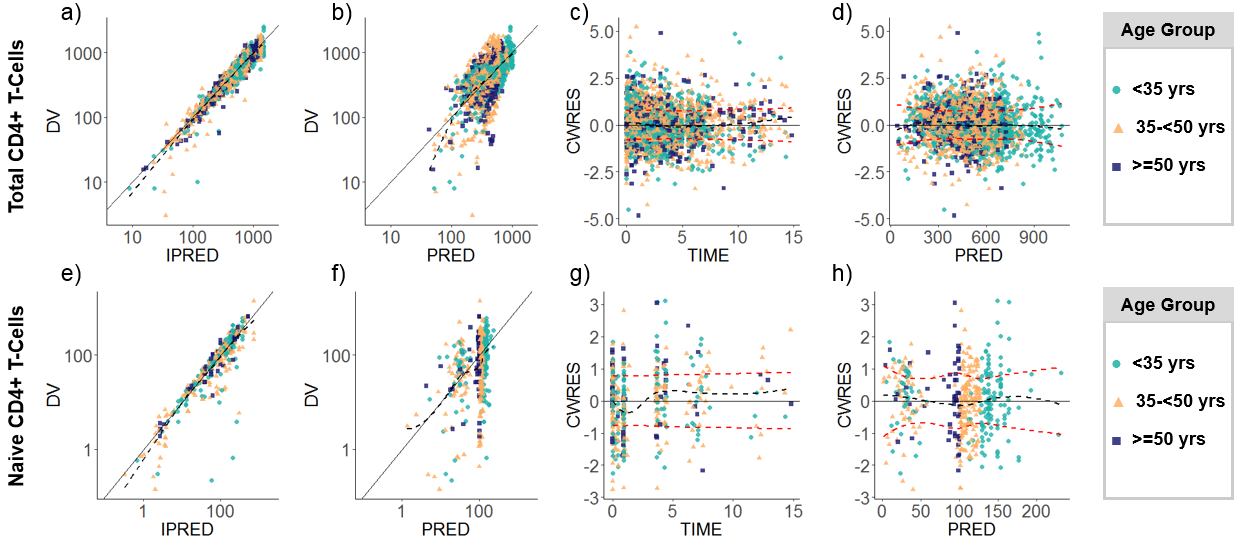


Supplemental Figure 4. Diagnostics of the Final Model.


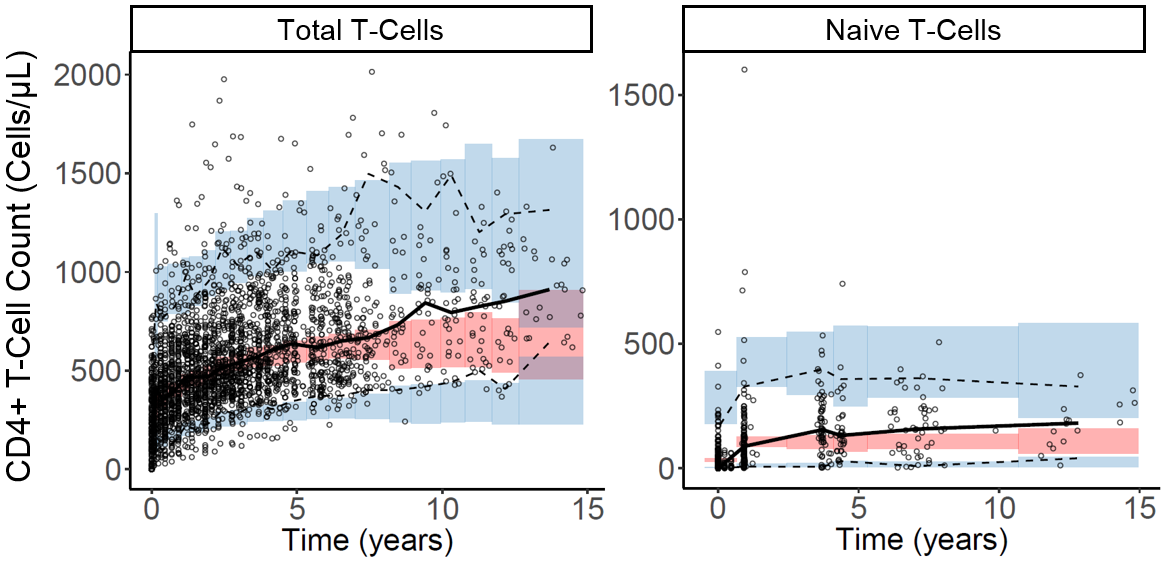


Black lines represent the observation percentiles: solid line for median and dot-dash lines for 5^th^ and 95^th^ percentile of observation. Black open circles represent the observation data. Colored shades are the 95% confidence intervals (CI) of the prediction percentile: red shades for 50% percentile and blue shades for 5^th^ and 95^th^ percentile.

Supplemental Figure 5. Prediction-Corrected Visual Predictive Checks (pcVPC) for CD4+ Total and Naïve T-Cells.


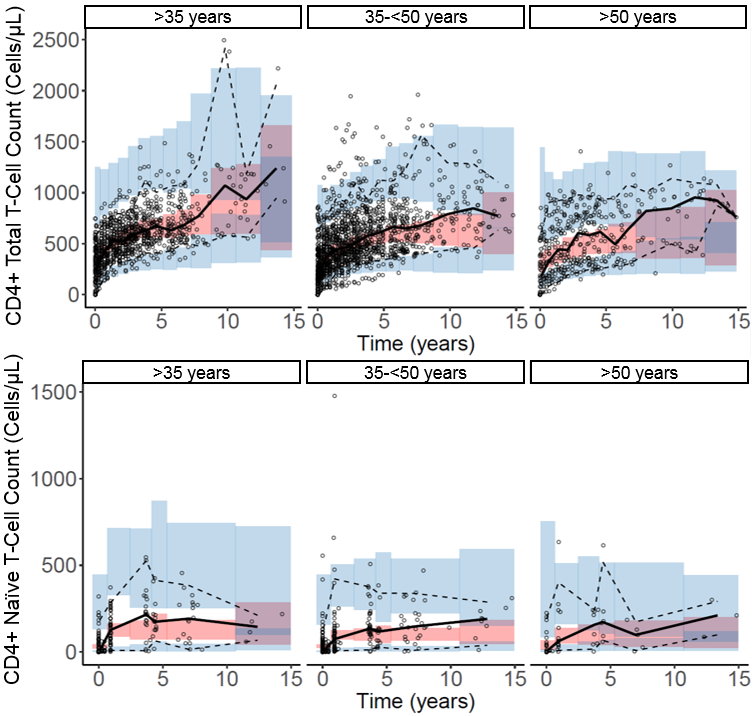


Black lines represent the observation percentiles: solid line for median and dot-dash lines for 5^th^ and 95^th^ percentile of observation. Black open circles represent the observation data. Colored shades are the 95% confidence intervals (CI) of the prediction percentile: red shades for 50% percentile and blue shades for 5^th^ and 95^th^ percentile.

Supplemental Figure 6. Prediction-Corrected Visual Predictive Checks Stratified by Age Group.

**Supplemental Table 2. Linear Regression Analysis of Post Hoc Parameters in the BLIR Study.**

|  | *σ* (cell/µL) • year^-1^ | | *d_N_* (year • cell/µL)^-1^ | | *α* (year^-1^) | | *d_M_* (year • cell/µL)^-1^ | |
| --- | --- | --- | --- | --- | --- | --- | --- | --- |
|  | Univariate | Adjusted | Univariate | Adjusted | Univariate | Adjusted | Univariate | Adjusted |
| Age at ART initiation | 0.7 | 0.93 | <0.001*** | <0.001*** | 0.62 | 0.89 | 0.97 | 0.57 |
| Sex | 0.64 |  | 0.87 |  | 0.84 |  | <0.001*** | <0.001*** |
| Baseline viral load | 0.88 |  | 0.29 |  | 0.58 |  | 0.19 |  |
| Smoking Status | 0.75 |  | 0.39 |  | 0.47 |  | 0.56 |  |
| BMI | 0.94 |  | 0.52 |  | 0.53 |  | 0.75 |  |
| %CD4+ T-cells from thymus | <0.001*** | <0.001**** | 0.38 |  | 0.12 |  | 0.36 |  |
| %Activated CD4+ T-cells | 0.92 |  | 0.94 |  | 0.59 |  | 0.19 |  |
| %Activated CD8+ T-cells | 0.24 |  | 0.034* | 0.11 | 0.24 |  | 0.36 |  |
| %Exhaustion | 0.16 |  | 0.83 |  | 0.022* | 0.030* | 0.7 |  |

**σ**: production rate of naïve T-cells, ***α***: activation rate constant of naïve T-cells into memory phenotype, ***d_N_***: apparent naïve T-cell elimination rate constant, ***d_M_***: apparent memory T-cell elimination rate constant, **BMI**: body mass index. Thymus output is defined by CD31+. Immune activation is defined by CD38+/HLA-DR+. Immune exhaustion is defined by PD-1+/TIM-3+. Log-transform of the data is applied when appropriate.

Significant codes: *** <0.001; ****** <0.01; ***** <0.05; **.** <0.1
